# Emerging Endemic Area for Blastomycosis, New York, USA, 2000–2024

**DOI:** 10.3201/eid3203.251306

**Published:** 2026-03

**Authors:** Laura E. Ramirez, Christian Kostowniak, Jessica Kumar, Sudha Chaturvedi, Ananthakrishnan Ramani, Amit Chopra

**Affiliations:** Albany Medical College, Albany, New York, USA (L.E. Ramirez, C. Kostowniak, J. Kumar, A. Ramani, A. Chopra); College of Integrated Health Sciences, State University of New York, Albany (J. Kumar); New York State Department of Health, Albany (J. Kumar); Wadsworth Center Mycology Laboratory, New York State Department of Health, Albany (S. Chaturvedi)

**Keywords:** blastomycosis, fungi, Blastomyces, endemic area, New York, United States

## Abstract

Blastomycosis is not yet considered endemic in upstate New York, USA; however, cases have increased during the past decade. We performed a retrospective study of 54 laboratory-confirmed cases reported during 2000–2024. Our results demonstrate an increase in incidence over time, indicating that this region represents an emerging endemic area.

The fungal infection blastomycosis had not been considered endemic to upstate New York, USA, but an increase in cases has been seen during the past decade (*1*,*2*), such that it could be an emerging endemic area (*3*). Despite increased efforts to raise awareness, this disease is not reportable in the state of New York. Because of the lack of recognition of blastomycosis as an emerging infection, diagnosis and treatment are often delayed (*3*). We conducted a retrospective study of patients with blastomycosis in this region, aiming to describe the epidemiologic characteristics and geographic distribution and to raise awareness of this disease.

## The Study

We retrospectively reviewed patients with diagnosed blastomycosis who were either hospitalized or managed in infectious disease clinics in Albany, New York, USA, during January 2000–December 2024. We identified patients with confirmed blastomycosis by reviewing pathology records, microbiology records, or both, through an electronic medical record system. We defined a confirmed case as a positive *Blastomycosis* spp. result for >1 of the following tests: real-time PCR, positive culture, or histopathology and cytopathology findings consistent with the diagnosis (*4*,*5*). Real-time PCR was performed on clinical specimens obtained directly from patients, including tissue biopsy specimens, bronchoalveolar lavage fluid, and sputum. The PCR used in this study was developed by the laboratory of the New York State Department of Health’s Wadsworth Center from BAD1 *Blastomyces* spp. with high sensitivity and specificity (*2*). The test has undergone the Clinical Laboratory Evaluation Program of the New York State Department of Health.

We calculated incidence of blastomycosis over 24 years. We used Tableau Desktop 2025.1.1 (Tableau, https://www.tableau.com) to generate a geographic map to visualize the incidence distribution across the affected regions using postal (ZIP) codes. We classified patients as having either isolated pulmonary infection or disseminated infection (involving >1 organ or any organ other than the lung). We compiled clinical characteristics, radiologic features, diagnostic methods, and treatment for both groups.

We identified 54 patients with a confirmed blastomycosis diagnosis during the study period (Table 1). Most (87%, n = 47) patients were hospitalized, but 7 (13%) patients were managed as outpatients. Nearly all cases were initially misdiagnosed, most commonly with community-acquired pneumonia or malignancy. More than half (57%) of patients had no identifiable underlying conditions (Table 1).

**Table 1:** Demographic characteristics and underlying conditions for 54 patients with laboratory-confirmed blastomycosis reported in Albany, New York, USA, 2000–2024*

Characteristics	Value
Mean age + SD, y	43 + 18
Median age, y (range)	42 (4–78)
Sex	
F	12 (22)
M	42 (78)
Average BMI + SD	27 + 6
Median BMI (range)	26 (16.9–39.7)
Race	
Caucasian	37 (69)
African American	9 (17)
Asian	2 (4)
Other	6 (11)
Underlying conditions	
Chronic lung disease	10 (19)
Diabetes mellitus	9 (17)
Cardiovascular disease	4 (7)
Malignancy	4 (7)
Immunosuppression	2 (4)
Organ transplant	1 (2)
HIV	1 (2)
Kidney disease and failure	2 (4)
Pregnancy	1 (2)
None identified	31 (57)
Tobacco use	30 (55)
Marijuana use	9 (15)
Preliminary diagnoses, n = 46	
Community-acquired pneumonia	14 (30)
Malignancy	12 (26)
Skin and soft tissue infection	5 (11)
Osteomyelitis	4 (9)
Bacterial and viral infection	3 (7)
Tuberculosis	1 (2)
Blastomycosis	1 (2)
Other	6 (13)

*Values are no. (%) except as indicated. Categories are not mutually exclusive, so totals may sum to >100%.

The most common diagnostic method for specimen collection was biopsy, performed in 42 (82%) patients. Biopsy was performed in 25/28 (89%) patients with disseminated infection and 17/23 (71%) patients with pulmonary infection. The most commonly biopsied organ was the lung (56%), followed by skin (25%) and bone (17%). Real-time PCR was used in 39 (78%) cases, culture in 29 (58%) cases, and histopathology/cytopathology in 13 (26%) cases. Only in 13/50 (24%) cases was PCR the sole method of diagnosis. More than 1 diagnostic method was used in 52% of cases (Table 2).

**Table 2:** Diagnostic evaluation and treatment patterns for patients with isolated and disseminated laboratory-confirmed blastomycosis reported in Albany, New York, USA, 2000–2024*

Characteristics	Isolated pulmonary	Disseminated	Total
Mean age + SD, y	45 + 17	40 + 19	43 + 18
Sex	n = 24	n = 28	n = 52
F	7 (29)	4 (14)	11 (21)
M	17 (71)	24 (86)	41 (79)
Method of specimen collection	n = 23	n = 28	n = 51
Biopsy	17 (71)	25 (89)	42 (82)
Bronchioalveolar lavage	14 (58)	3 (11)	17 (32)
Sputum	2 (8)	1 (4)	3 (6)
Lumbar puncture	0	3 (11)	3 (6)
Methods of establishing diagnosis	n = 22	n = 28	n = 50
Real-time PCR	17 (77)	22 (79)	39 (78)
Culture	12 (55)	17 (61)	29 (58)
Histopathology and cytopathology	6 (27)	7 (25)	13 (26)
Computed tomography chest characteristics	n = 24	n = 12	n = 36
Mass	8 (33)	5 (42)	13 (36)
Lobar consolidation	9 (38)	3 (25)	12 (33)
Nodular	7 (29)	4 (33)	11 (31)
Pleural effusion	3 (13)	3 (25)	6 (17)
Mediastinal lymphadenopathy	2 (8)	2 (17)	4 (11)
Cavity	3 (13)	0	3 (8)
Miliary	1 (4)	1 (8)	2 (6)
Treatment	n = 19	n = 21	n = 40
Itraconazole only	13 (68)	11 (53)	24 (60)
Amphotericin B followed by itraconazole	4 (21)	6 (29)	11 (28)
Amphotericin B only	2 (11)	2 (10)	4 (10)
Fluconazole	0	2 (10)	2 (5)
Voriconazole	1 (5)	0	1 (3)
Length of treatment, n = 31	n = 15	n = 16	n = 31
6 mo	7 (47)	1 (6)	8 (26)
9 mo	0	1 (6)	1 (3)
12 mo	8 (53)	14 (88)	22 (71)
Death	1 (2)	2 (4)	3 (6)
Total	24 (46)	28 (54)	52

*Values are no. (%) except as indicated. Categories are not mutually exclusive, so totals may sum to >100%. Data were not available for all variables for every patient; therefore, denominators vary by category. Percentages are calculated by using the number of patients with available data for each variable.

**Figure 1:**
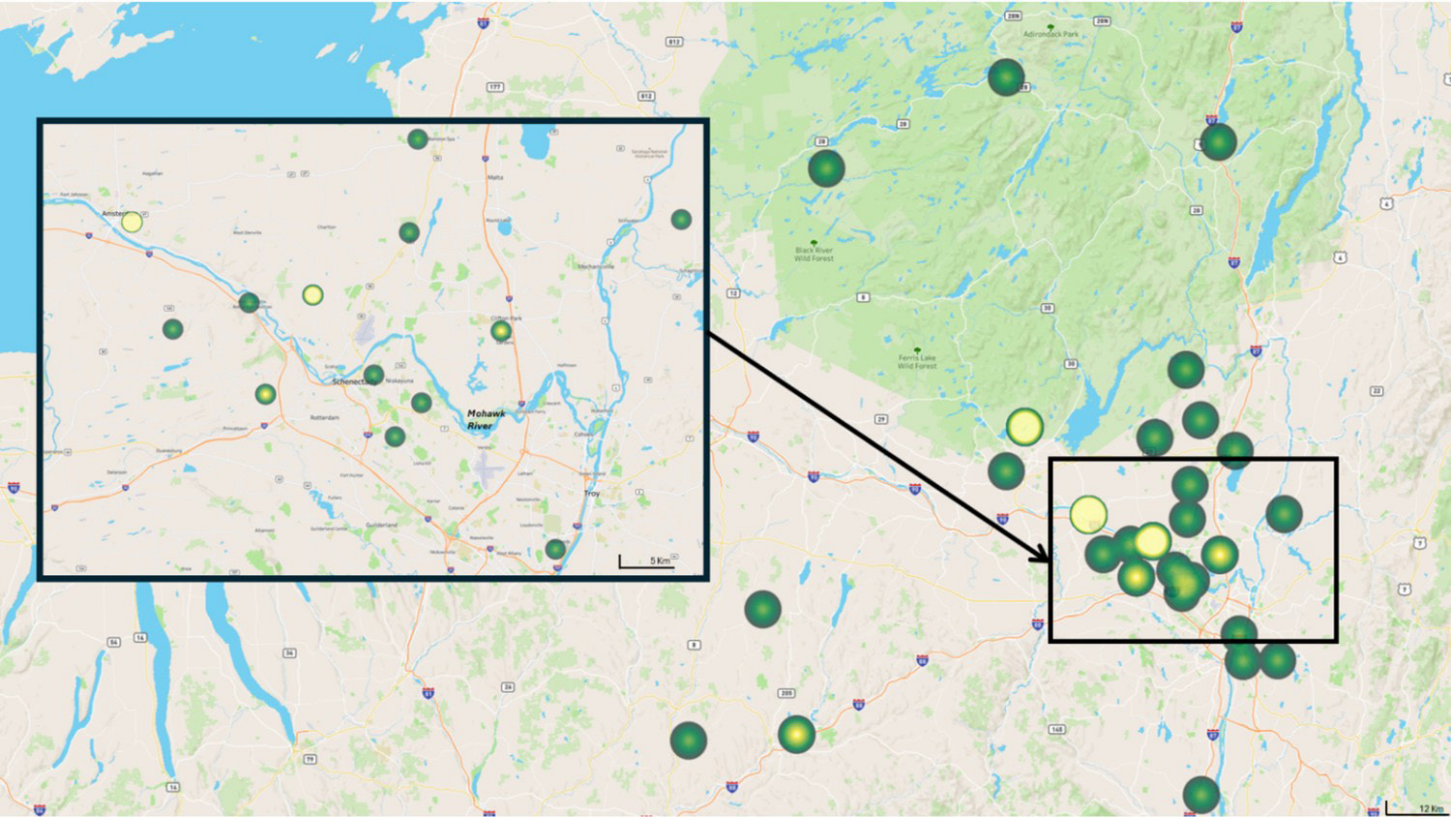
Geographic distribution of blastomycosis cases in upstate New York, USA, 2000–2024. Yellow shading indicates a higher number of cases associated with that postal (ZIP) code. A higher concentration of cases is observed in regions near the Mohawk River (inset), suggesting a potential area of increased endemicity.

**Figure 2:**
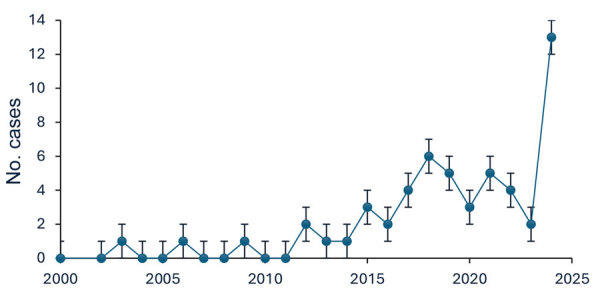
Annual number of blastomycosis cases diagnosed at Albany Medical Center, Albany, New York, USA, 2000–2024. Case numbers remained stable during 2000–2014, then markedly increased during 2015–2024. Error bars indicate SE.

Most (96%) patients were residents of upstate New York, and 85% of those lived in counties within the Capital District region around Albany (Appendix Figure). No patients reported travel to known endemic areas. We mapped the geographic distribution of cases along the Mohawk River (Figure 1). The number of reported blastomycosis cases rose substantially during the study years, but the most pronounced rise occurred in 2024, which represented 24% (13/54) of total cases (Figure 2). Those cases were distributed over multiple counties in the region and not concentrated in 1 area.

Disseminated infection was present in 54% of patients; the other 46% had isolated pulmonary involvement. We compared the clinical characteristics, radiologic features, diagnostic methods, and treatments for those 2 groups (Table 2) and found no noticeable differences. We did not perform statistical analyses for the comparison of the 2 groups because of the small sample sizes.

The results from this study demonstrate the recent increase in the incidence of blastomycosis cases in upstate New York. Many patients resided near the Mohawk River Valley area, highlighting the disease’s rising incidence in that region. None of the patients in this study had documented travel history to known endemic areas, implying that the infection was acquired locally. That increasing trend might be the result of climate change causing a more favorable environment for the growth or sporulation of *Blastomyces* species. Previous studies have suggested that a global increase in temperature and rainfall has played a key role in the rising number of blastomycosis cases and its spread to nonendemic areas, given that the fungus thrives in moist and warm environments (*6*,*7*). Similarly, temperatures and precipitation in upstate New York have risen over the years (*8*), contributing to a more favorable environment for *Blastomyces* spp. growth. Another factor that might have contributed to the increasing trend is increased disease detection with PCR, which has high sensitivity and specificity for *Blastomyces* spp. (*9*).

Historically, blastomycosis was recognized as endemic in parts of North America, particularly in regions surrounding the Ohio and Mississippi River Valleys and the Great Lakes (*10*). The infectious etiology in this study follows a similar pattern, with a high incidence of cases reported in areas along the Mohawk River (Figure 1). Currently, only 5 US states require reporting blastomycosis to the health department: Arkansas, Louisiana, Michigan, Minnesota, and Wisconsin (*11*). The cases described in this study reflect a single medical center, suggesting that the data are probably a fraction of the actual disease burden in the region and that the true incidence might exceed official estimates. In 2017, the Centers for Disease Control and Prevention, alerted by the New York State Department of Health, reported an increase in blastomycosis incidence in the Capital District region among patients with no travel history to endemic areas (*12*). Other studies have provided evidence of the increasing blastomycosis incidence, suggesting that upstate New York could be an emerging endemic region (*3*,*13*,*14*).

Diagnosis of blastomycosis requires a high index of clinical suspicion, particularly in patients with pulmonary infections and extrapulmonary lesions. Such patients often have no notable underlying conditions. Given that nearly all patients in this cohort initially had misdiagnoses and that New York is not currently considered an endemic region, most patients experienced a delay in diagnosis and appropriate treatment.

The first limitation of this study was its single-center retrospective nature, which limits the generalizability of our results and creates the need for further studies with a broader sample of the population. Second, asymptomatic patients were less likely to seek medical care and were thus omitted. Third, PCR testing was performed on samples from many patients, but PCR is not widely available at other hospitals. However, in many cases, results were also confirmed by culture results. Fourth, some patients did not have comprehensive clinical and diagnostic data available for a thorough analysis. Last, some patients might have received treatment at a different medical center and were not captured in this analysis. Therefore, the cases we report do not represent the true incidence of cases in the community, although results were validated with the state public health laboratory at the Wadsworth Center.

## Conclusions

Despite the limitations, our results strongly indicate that upstate New York represents a new emerging endemic area for blastomycosis. Therefore, we recommended that this disease be made reportable to the state’s health departments. Physicians in this region should consider blastomycosis when pulmonary symptoms are accompanied by cutaneous findings, potentially reducing initial misdiagnoses and treatment delays.

Appendix Additional information for emerging endemic area for blastomycosis, New York, USA, 2000–2024.
