# Supplementary material for: Emerging Endemic Area for Blastomycosis, New York, USA, 2000–2024
Source: Emerg Infect Dis. 2026 Mar;32(3):414–8. doi: 10.3201/eid3203.251306 (PMC13016025; doi:10.3201/eid3203.251306)
Supplement: Appendix — Additional information for emerging endemic area for blastomycosis, New York, USA, 2000–2024. [file 25-1306-Techapp-s1.pdf]

Article DOI: https://doi.org/10.3201/eid3203.251306

EID cannot ensure accessibility for supplementary materials supplied by authors. Readers who have difficulty accessing supplementary content should contact the authors for assistance.

Emerging Endemic Area for Blastomycosis, New York, USA, 2000–2024

Appendix

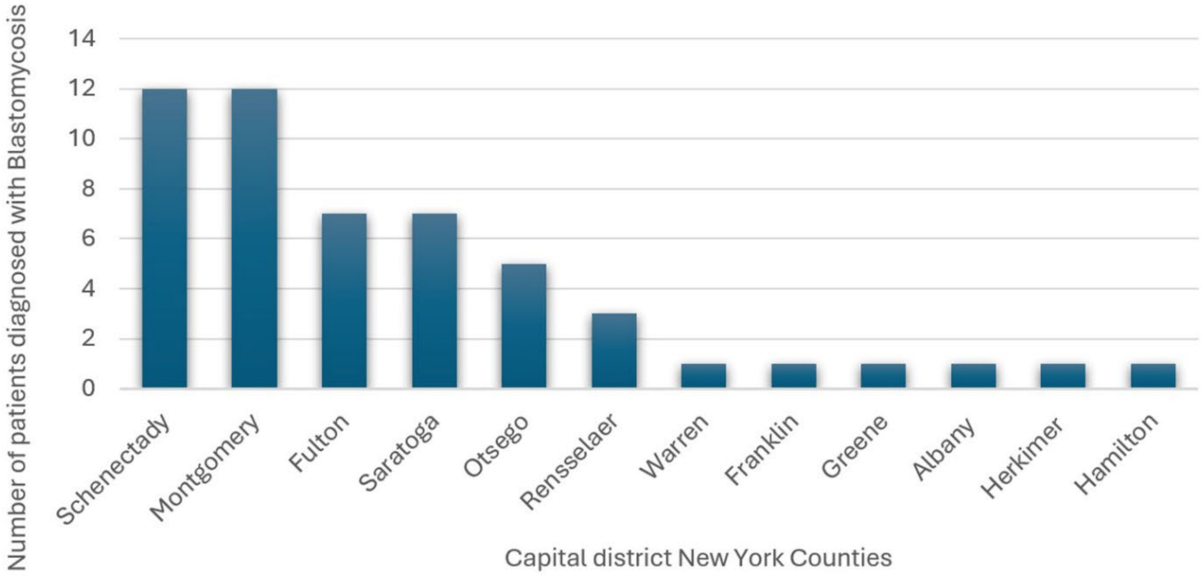

Appendix Figure. Distribution of blastomycosis cases across counties in study area of the Capital Region of New York, USA.
